# Engaging stakeholders to identify gaps and develop strategies to inform evidence use for health policymaking in Nigeria

**DOI:** 10.11604/pamj.2022.43.140.36754

**Published:** 2022-11-15

**Authors:** Ejemai Eboreime, Oluwafunmike Ogwa, Rosemary Nnabude, Kasarachi Aluka-Omitiran, Aduragbemi Banke-Thomas, Nneka Orji, Achama Eluwa, Adaobi Ezeokoli, Aanu Rotimi, Laz Ude Eze, Vanessa Offiong, Ugochi Odu, Rita Okonkwo, Chukwunonso Umeh, Frances Ilika, Adaeze Oreh, Faith Nkut Adams, Ikedichi Arnold Okpani, Yewande Ogundeji, Chinyere Mbachu, Felix Abrahams Obi, Okikiolu Badejo

**Affiliations:** 1 Systems Development Initiative, Abuja, Nigeria,; 2 Department of Psychiatry, University of Alberta, Edmonton, Canada,; 3 Doctorkk Health International, Lagos, Nigeria,; 4 School of Public Health, University of Alberta, Edmonton, Canada,; 5 Department of Community Health Services, National Primary Health Care Development Agency, Abuja, Nigeria,; 6 School of Human Sciences, University of Greenwich, London, United Kingdom,; 7 London School of Economics and Political Science, London, United Kingdom,; 8 Department of Health Planning, Research and Statistics, Federal Ministry of Health, Abuja, Nigeria,; 9 Health, Nutrition and Population Global Practice Unit, The World Bank, Washington DC, United States of America; 10 Harvard Kennedy School, Harvard University, Cambridge, Massachusetts, United States,; 11 Centre for Accountability and Inclusive Development, Abuja, Nigeria,; 12 Talk Health Real Media Limited, Abuja, Nigeria,; 13 As Equals, CNN International, Abuja, Nigeria,; 14 Healthreach limited, Abuja, Nigeria,; 15 Institute of Human Virology Nigeria, International Research Center of Excellence, Abuja, Nigeria,; 16 African Youth Initiative on Population Health and Development (AfrYPoD), Abuja, Nigeria,; 17 Palladium, Health Policy Plus Project, Abuja, Nigeria,; 18 Department of Planning, Research and Statistics, National Blood Transfusion Service, Abuja, Nigeria,; 19 Direct Consulting and Logistics, Abuja, Nigeria,; 20 School of Population and Public Health, The University of British Columbia, Vancouver, Canada,; 21 O'Brien Institute for Public Health, University of Calgary, Calgary, Canada,; 22 Health Policy Research Group, College of Medicine, University of Nigeria, Enugu, Nigeria,; 23 Results for Development (R4D), Nigeria Country Office, Abuja, Nigeria,; 24 Department of Public Health, Institute of Tropical Medicine, Antwerp, Belgium

**Keywords:** Knowledge translation, stakeholder engagement, policymaking, health system, Nigeria

## Abstract

**Introduction:**

recent efforts to bridge the evidence-policy gap in low-and middle-income countries have seen growing interest from key audiences such as government, civil society, international organizations, private sector players, academia, and media. One of such engagement was a two-day virtual participant-driven conference (the convening) in Nigeria. The aim of the convening was to develop strategies for improving evidence use in health policy. The convening witnessed a participant blend of health policymakers, researchers, political policymakers, philanthropists, global health practitioners, program officers, students, and the media.

**Methods:**

in this study, we analyzed conversations at the convening with the aim to disseminate findings to key stakeholders in Nigeria. The recordings from the convening were transcribed and analyzed inductively to identify emerging themes, which were interpreted, and inferences are drawn.

**Results:**

a total of 630 people attended the convening. Participants joined from 13 countries. Participants identified poor collaboration between researchers and policymakers, poor community involvement in research and policy processes, poor funding for research, and inequalities as key factors inhibiting the use of evidence for policymaking in Nigeria. Strategies proposed to address these challenges include the use of participatory and embedded research methods, leveraging existing systems and networks, advocating for improved funding and ownership for research, and the use of context-sensitive knowledge translation strategies.

**Conclusion:**

overall, better interaction among the various stakeholders will improve the evidence generation, translation, and use in Nigeria. A road map for the dissemination of findings from this conference has been developed for implementation across the strata of the health system.

## Introduction

The field of knowledge translation (KT) within Health policy and systems research (HPSR) in low- and middle-income countries (LMICs) is advancing. Knowledge translation has sought answers to bridge the evidence-policy gaps that exist between researchers (knowledge producers) and policymakers (knowledge users) who (are expected to) use research-produced knowledge to make health policies [1-3]. Within this space, support for evidence-informed policy making has gained traction in recent years, but consensus around the topic is by no means a given [4-7]. For example, arguments have been put forward that KT approaches in evidence-based medicine would not work in public health policy making [8]. Various actors have also debated what constitutes “evidence” in policymaking. One article suggests that evidence can be viewed through three lenses: systematic ('scientific') research, program management experience ('practice'), and political judgement [9]. Thus, dissimilarities in values, worldviews, and knowledge between policy makers and researchers serve as a roadblock to effective bidirectional communication and appreciation of evidence use in policy making [1,5,10]. Consequently, scientific evidence is often relegated as less important in the policymaking space. This is concerning as there is an ever-increasing need for more evidence-based or evidence informed policies across the world. More than ever before, the COVID-19 pandemic raised new and important questions about the relevance of scientific research evidence in the context of highly politicized health issues and socio-political environments. Following years of apparent progress in promoting the uptake and utilization of evidence in policy making, the pandemic has demonstrated that the production of scientific evidence is a necessary but not sufficient ingredient to promote evidence-informed policymaking [11-13]. For example, despite the evidence that optimal vaccination rates across all countries would be a significant mitigator of the pandemic thus should be a priority pursuit, many high-income countries have resorted to vaccine hoarding and border restrictions which are far less effective and could pose economic challenges[14-16]. Similarly, in many LMICs like Nigeria, local epidemiological and social science evidence was ignored by policymakers in favor of contextually ineffective lockdowns, which were excessively coercive yet with minimal compliance[11,13].

Knowledge translation and HPSR are still relatively new and evolving in Nigeria but are already regarded as priority research need. Health policy and systems research is identified in the National strategic health development plan as one of the eight priority areas that aim to utilize research to inform policy and programs, improve health and contribute to the global knowledge platform [17]. Whereas there has been significant growth in HPSR in Nigeria over the past 20 years, there has not been a commensurate translation of this knowledge expansion into the policy space. The limited use of research findings by policymakers and communities may be partly explained by inadequate and insufficient capacity to produce and use HPSR [1,10,18]. Recent efforts to promote HPSR as a means to bridge the evidence-policy gap in low- and middle-income countries has seen growing interest from key audiences such as government, civil society, international organizations, private sector players, academia and media [1,19-23]. Nigeria is not an exception, and this development signifies that a broader range of policymakers in the country are becoming potential evidence-users´ than ever. It is therefore important to take stock of what is known and what is not yet known about evidence-based policy in the country, and what can be done to assist the growing stakeholder base. Part of this process would necessarily entail a comprehensive look at researchers´ and policy makers´ mutual perceptions, understanding whether and how conditions related to evidence and policy have changed in recent years and to identify new recommendations for a socio-political context characterized by uncertainty over the value of evidence. Recognizing that evidence-policy gap, and the required interventions to bridge the gap vary between settings, we set out to engage stakeholders in Nigeria to identify gaps and develop strategies for improving evidence use in health policy. The engagement was conducted as part of the convening supported by Health systems global (HSG) and implemented by the systems development initiative in 2020. The aim of the engagement was to develop strategies for improving evidence use in health policy. In this study, we evaluated the conversations by stakeholders during the convening. Our objective in this study was to understand multi-stakeholder perceptions of the role of research evidence in the policy making process. Such findings and recommendations are important to a variety of stakeholders, including national and sub-national level government agencies and entities, research funders, donors, knowledge-producing entities like universities and university-based centers that fund and promote research, and intermediary organizations, such as professional societies for researchers and practitioners

## Methods

**The convening:** the convening was a two-day virtual participant-driven conference. The online virtual approach was designed to address the need to adhere to the public health protocol of social distancing considering the COVID-19 pandemic at the time of the event. Hence, the event adopted a combination of different formats to build community, unleash initiatives, and help solve problems, while also creating an enabling mechanism for effective participation and interrogation of health policy-related issues. These formats included but were not limited to, plenary sessions, with attendant breakout sessions, panel discussions led by seasoned practitioners, academics, and policymakers vast in the field of HPSR with adequate Question and Answer sessions after each panel discussion. The convening lasted for a maximum of six hours spread over the two days of the event. Publicity for the event was launched over 40 days before the due date. We adopted an online media mechanism via social media platforms (Twitter, Instagram, Facebook, WhatsApp, etc.) as well as conventional mainstream media like radio and print media in disseminating information about the convening. The team reached out to a wide scope of knowledge-based bodies, academia and relevant institutions that play an active role in health policy research within the African continent. The list of organizations we partnered with is as detailed in the Table 1. Our approach encompassed activities that preceded and continued beyond the convening to ensure continued conversations and learnings (see work stream below). This sustained advocacies and other efforts that this convening stimulated and, more importantly, dovetailed into HSR2020 and HSG overall agenda for improved HPSR capacity building.

**Table 1:** characteristics of participants

Profession	Number	%
Academic/ researcher	86	13.65%
Civil society/ faith-based organization	23	3.65%
Development worker/donor agency	228	36.19%
General public	10	1.59%
Healthcare professional (frontline)	144	22.86%
Intern/student	42	6.67%
Journalist	9	1.43%
Policy maker/government	53	8.41%
Private Sector	35	5.56%
Total	630	100.00%

**Convening participants:** the convening witnessed a participant blend of health policymakers, researchers, political policymakers, philanthropists, global health practitioners, program officers, students and, the media drawn from diverse relevant audiences including Federal ministries, departments, agencies, and academia (Table 1). Methods of invitation included but were not limited to official letters of invitation and online registration portals.

**Data analysis:** the zoom audio-visual recordings were sourced from YouTube where they are publicly available [24]. The data was transcribed by Rosemary Nnabude. Inductive thematic analysis, proposed by Braun and Clarke [25], was employed involving two analysts (Ejemai Eboreime, Oluwafunmike Ogwa). The authors familiarized themselves with the transcribed and audiovisual data. Thereafter, preliminary codes were ascribed to the data in order to describe the content. The analysts searched for patterns and emerging themes independently. Thereafter, these themes were collectively reviewed, and the final themes were defined and named.

**Ethics approval and consent to participate:** ethics waiver was obtained from the Nigerian National Health Research Ethics Committee (NHREC) to conduct this study with approval number: NHREC/01/01/2007-15/11/2021.

## Results

**Figure 1:**
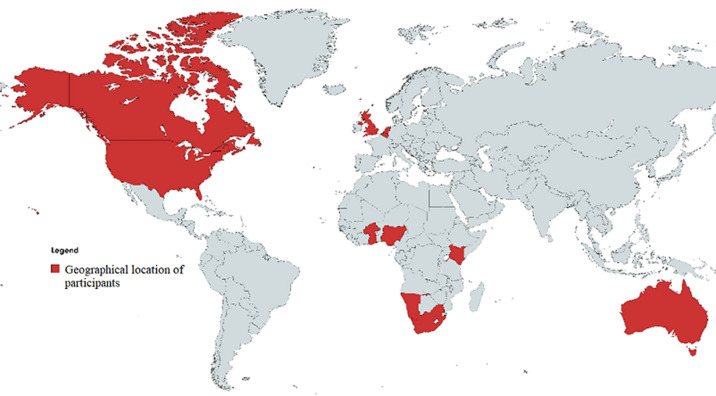
geographical distribution of participants

**Participants:** a total of 630 people were in attendance at the convening. Participants joined in from 13 countries (Figure 1). The majority (491 (78%) participated from Nigeria. This is followed by 75 (12%) participants from the United States, 25 (4%) from the United Kingdom and Canada respectively, two participants from Burkina Faso and South Africa respectively, while other countries highlighted had one participant each. 302 (48%) of the attendees were females. 53 (8.4%) identified as policy makers or government officials, 86 (13.7%) were researchers, frontline health workers 144 (22.9%), civil society made up 23 (3,7%), 42 (6.7%) were students, while most participants (228 (36%) identified as working with development or donor organizations.

**Figure 2:**
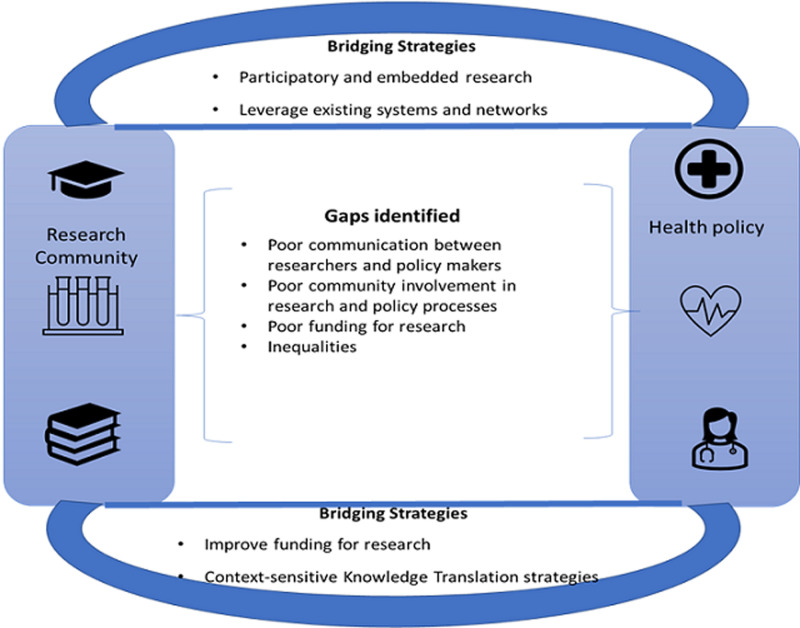
summary of outcomes (gaps and bridging strategies)

**Outcomes of the discussions:** in Figure 2 is an illustrative summary of the gaps identified with respect to utilization of evidence in health policy, as well as strategies proposed to bridge this gap. Barriers to evidence-informed policymaking. The barriers to the generation and routine use of evidence in health policy and systems decision making were highlighted by participants.

**Poor collaboration between researchers and policymakers:** participants identified a disconnect between the policy agenda and the research agenda. They opined that policy needs or policy-relevant questions do not often inform research being conducted. Consequently, policymakers do not seek available research because it does not address the questions or needs of the policymaker or “real” implementation problems. If collaborations were improved, the research or research agenda would be informed by policy needs. Such collaborations will make research findings more interesting or useful to policymakers. Participants also identified lack of awareness of existing or available evidence by decision-makers, lack of motivation to appraise research findings a lack of accountability as major barriers. Whereas there is a department of planning, research, and statistics (PRS) in the Ministry of Health and agencies, much of the work being done in these PRS departments had to do with planning and collation of statistics (data). But the research component was largely dormant. One panelist from academia called this “the missing R”. This gap was largely created by poor capacity for research, poor funding, and the lack of demand for evidence in the policy space. The panelist opined that revitalizing the missing R should be a priority for policymakers. This process would require active engagement with the research community. Another panelist (a policymaker) in agreeing with this position, opined that lots of the research being generated by academics were often not relevant to policymaking. Policymakers often find research difficult to understand or apply because it lays more emphasis on scientific language. Appropriate knowledge translation mechanisms co-developed by all categories of stakeholders would improve the usability of the research findings in the policy space. Greater involvement of researchers in policy space and vice versa was advocated. This can be achieved through coproduction and other collaboration techniques. *"There is a gap between researchers and policymakers in Nigeria as a result of political and religious factors as well as policy and scientific uncertainties. The relevance of research finding to a particular context is sometimes missing with respect to risk conception and timing. Often, by the time research is concluded, what it was designed to inform would be over"*(policymaker).

**Poor community involvement in research and policy processes:** participants recognized that users of health were often neither actively represented in policymaking nor evidence generation for policy. Neither research nor policy decisions utilized participatory methods, even though community governance structures such as the Ward Development Committees were existed yet not empowered. In considering policy formulation and research, the government, researchers, Civil Society Organizations (CSOs) were identified as essential in ensuring that communities are involved through the whole spectrum of policy development. *"Politicians make the ultimate decisions to advance their own interest, donors have their own interest, implementers even have their own and the general populace have theirs as well. We can address the problem of community engagement through the CSOs. Nobody is inclined to give up their interest for the people except the ones who represent the people and therefore fight for their people´s interest. CSOs also need the capacity to appraise evidence and inform their advocacy so as not to advocate for the wrong things. We cannot solve people´s problems without viewing issues from their perspective"*(civil society). Some topics that also came up during the deliberations include reflections on the extent to which the CSOs actually represent the needs and desires of the communities. It was recommended to identify CSOs that have strong mechanisms in place for eliciting the citizen's voices and desires. *“Sometimes, coproduction meetings for research may involve direct consultation of direct users or beneficiaries in the communities outside the traditional CSO organizations to get a deeper insight into more meaningful research.”* (development worker).

**Poor funding for research:** research is poorly funded in Nigeria. There is very minimal government funding for research institutions. These mostly come in the form of salaries for university staff and infrastructure. Similarly, the annual health budget often captures little or no research component. Most of the funding for research in Nigeria is from donor sources. The strong reliance on donor funding for research and development is reflected in the influence of donors on national research priorities and policy decisions. Dependence on donor funding weakens local participation and ownership in knowledge production. *"We have to make sure we have our own house in order. We need country ownership and leadership. If this is not in place, whenever a donor comes, we simply agree to their agenda without appropriate direction"* (researcher). *"Foreign researchers perform research in Nigeria many times without engaging the local researchers. The findings are published in subscription-based journals which may not be accessible to policymakers nor resonate with them when accessible as a result of the scientific jargon used"*(researcher).

**Gender inequality:** gender inequality was the most discussed inequality at the convening. The existing challenges with the placement of women in influential positions in HPSR were enumerated. These include not recognizing the impact of women as domestic implementers of health policy, as well as exclusion from their roles by a society that does not promote women in leadership. Women are much more involved with the informal level of the health system. They are usually the first responders and take responsibility for health challenges in the home and community. Yet, as noted by participants and panelists, women are often excluded from the decision-making space at home and in society, including health policymaking at the community and higher levels. Sociocultural barriers often lead to inequalities in education for the girl child. Further, women are often less preferred than men for strategic jobs in policymaking, even when better qualified. Including women in decision making from the grassroots level upwards will likely improve the uptake of evidence, given that women are often essential subjects of most community health research, but made the objects of decision making. *"We already have a good system for women at the grass-root level of our health system, but as they rise up the pyramid, they begin to fall off and we have a testosterone festival at the top. It is a pyramid with women at the bottom. Poor engagement of women in the network of governance is a reason why many policies fail in the system"* (researcher). *"Households, where mothers take part in decision making, are actually more susceptible to better family health outcomes we would expect better appreciation of evidence in policy with the engagement of women, who traditionally interact more with the health system than their male counterparts”* (civil society). In order to systematically address the existing gender barriers, the need for contextualizing the strengthening of health systems by including gender analysis in HPSR as well as putting gender-specific measures in place to advance the capacity of men and women was emphasized. *"Gender-related problems should be addressed as man, woman and transgender issues. Identifying champions of gender issues who can communicate in a gender-specific manner and promote gender equality is important." (Researcher)*

**Transgenerational inequalities:** participants also noted that transgenerational inequalities exist as barriers to evidence-informed policymaking. Younger people are not commonly involved in academic research or in policy spaces, even though Nigeria has a very large youth population. The need to build local capacity for research, as well as actively ensure that young people are represented at the high-level decision spaces, was emphasized. *“The older generation dominate the universities and also the government. Decisions about youth are often made by older people. This is a major reason why policies fail”* (development expert). *“Training on rigorous research is weak in Nigerian educational programmes. Many Nigerians who have acquired relevant skills did so independently of the formal system in Nigeria. Some had to go to school abroad. Many of these never return. Those who return are not given the opportunity to utilize their newfound knowledge in the universities or in government”* (student).

**Strategies to bridge the gap:** strategies highlighted by participants for promoting the use of HPSR in Nigeria were capacity building of decision-makers to understand the principles and usefulness of HPSR; capacity building of researchers to enable them to undertake useful and relevant research; developing a framework within organizations and ministries of health on how to apply and utilize the available evidence; provision of structures that build trust and relationship between researchers and policymakers.

**Participatory and embedded research:** the need to develop linkages among stakeholders including CSOs, health care providers, academia, policy implementers makers and implementers as well as donors and partners were noted to be essential for translating research into policies and policies into action. Participatory and embedded research methods including co-production of research topics and agenda were recommended. *"Academic researchers should not develop research questions in silos. Rather the needs of the community should be addressed by ensuring a conscious approach in developing a solid research agenda. There is a need for a feedback loop through all the mechanisms to ensure that evidence is rightly interpreted and utilized"*(researcher). The entry points for action for people who work in HPSR at strengthening the conduct of research that is more focused on policy in Nigeria were enumerated by participants to include embedding researchers in the process of policymaking and knowledge brokering. *"We need to work with intermediaries who can help strengthen relationships and networks. They will also source for knowledge for policymakers and communicate to researchers where the interest of the policymakers lies" (researcher)*.

**Leveraging existing systems and networks:** it was agreed that the scale-up of evidence in HPSR in Nigeria could be achieved by utilizing the available platforms to disseminate credible information that would be scaled up across the country. These platforms include the Nigeria governors´ forum (NGF), the Legislative network for universal health coverage, Committee of Health Commissioners and CSOs. In addition, a need for an academic publishing infrastructure was identified as a means of scaling evidence in Nigeria across all the States. *"Bridge the gap between decision-makers and researchers by establishing postdoctoral positions in the policy-making space. Create policy liaison units in all ministries that will harness evidence and disseminate same to relevant offices so that the evidence can be appropriately utilized. It is important to have a clear learning research agenda that includes the highlights of key policy issues encountered by policymakers. There is a need for improved capacity building in implementation research" (researcher)*.

**Improve funding for research:** while improving the grantsmanship skills was noted as an important capacity for both the academic and policymaking communities, ensuring the sustainability of research funding in Nigeria would require the government to take ownership of HPSR. Government can do this by developing an interest in knowledge production and consequently prioritizing research funding. The Federal Government´s Tertiary Education Trust Fund (TETFund) was identified as an institution that could be used by the government to provide funding for health policy and systems research. It was also suggested that the directorates of planning, research, and statistics (DPRS) at the national and State levels in relevant ministries should provide for research budget lines in the annual government budgets. Further, it was recommended that the DPRS develop yearly research agenda and plans collaboratively with the earlier mentioned stakeholders (researchers, CSOs, policymakers) that encompass the needs of communities, policy implementation challenges and policy burning questions. This research agenda should be made publicly available on websites and channels that researchers and other stakeholders can access to inform their research. This would demonstrate government ownership of evidence to policy process, and result in less dependence on donors for essential health policy research. *"It is more sustainable to use our government money for research. We are in the era of development corporations, no longer in the donor era." ''TETFund can issue calls for proposal to universities to conduct HPSR while establishing a loop to communicate the research findings back to the ministry of health" (researcher)*.

**Adopt context sensitive knowledge translation strategies:** the significance of disseminating existing actionable evidence to the relevant group of people for effective utilization through appropriate platforms was emphasized. Decision-makers are sometimes provided with relatively technical and complex information that may not be adequately comprehended. It was stated by participants that relevant data is what should be made available and accessible to policymakers in a way that can be understood by them. These avenues include policy briefs, blogs and short videos while paying attention to the appropriateness and sensitive nature of the evidence to the political class. Further, it is essential that conscious effort be made to build into research from the onset, what should be the endpoint of the research beyond the dissemination of research findings to how the findings will be used to influence policy and implementation. *"We need to think more about adaptation and contextualizing because Nigeria has not one health system, but thirty-seven health systems*" (policymaker). *"It is very important for us to think and work politically while presenting evidence to understand what the politicians are sensitive to. We need to know how to engage them and incorporate in our learning and research agenda how to engage and communicate effectively with them" (researcher)*.

## Discussion

Significant work has been done over the years to bridge the gap between research and policymaking in Nigeria. These efforts have resulted in considerable improvement [2,19,20,26-29]. However, our study (as many other studies) shows that more work needs to be done to improve the use of evidence in health policymaking. Our analysis of data from the convening found that participants identified poor collaboration between researchers and policymakers, poor community involvement in research and policy processes, poor funding for research and inequalities as key factors inhibiting the use of evidence for policymaking in Nigeria. Strategies proposed to address these challenges include the use of participatory and embedded research methods, leveraging existing systems and networks, advocating for improved funding and ownership for research, and the use of context-sensitive KT strategies by means of policy briefs, infographics, among others.

**Collaborations among health system actors:** globally, a collaboration between policymakers and researchers remains weak [1,30]. As our study revealed, the research-policy space is not different in Nigeria. Some models that have been applied to bridge this gap include policy dialogues, two-way secondment, building the research capacity of policymakers, among others [26-28]. The two-way secondment model initiated by Uneke and colleagues at the subnational level is a unique strategy that may yield effective results if scaled up. The model involves the posting of researchers to the Ministry of Health to contribute to policymaking, as well as learn policy-making processes. Similarly, policymakers are seconded to universities and other research institutions to learn evidence generation, as well as to provide insights to researchers on how policies are made in the real world. Implementation of this strategy led to a measurable increase in knowledge of policy analysis and contextualization among the seconders up to 50% [27]. Among other strategies, this two-way secondment model may be effective in addressing the problem of the “missing R” in policymaking as pointed out by discussants in the convening.

Community involvement: community involvement in decision-making is key to effective policies, as discussed during the convening. The exclusion of the populace in policy processes is not a new phenomenon across the world, irrespective of advancement in democracy [31]. But putting the people at the center of evidence production in health policy research is an emerging paradigm. There is abundance of evidence that community involvement in policy processes can significantly improve the use of evidence in policymaking[32]. Whereas the well-known Caplan´s “Two-communities theory” focuses on KT between researchers and policy makers, it is increasingly becoming recognized that more than two communities exist for effective KT. Thus, the emergence of people-oriented research methods such as participatory action research (PAR) and embedded research [3]. There is now recognition that people are not mere objects of research (such as being respondant to data collection), but partner researchers [1,10]. Bottom-up decision-making models are being evolved for policy making in many sub-Saharan African countries [33]. For example, Nigeria has used an iterative bottom-up model in which communities, policymakers and researchers were involved in iterative evidence generation and planning to improve the coverage of primary health care [34-37]. In post-Ebola Liberia, people-centered maternal health services have been evolved using PAR and embedded research methods [38]. These strategies can break the trichotomy among researchers, policymakers, and health service users as well as address systemic inequalities.

**Funding for evidence generation and translation:** funding is central to the generation and use of evidence, as emphasized by participants in the convening. Many low- and middle-income countries struggle to finance activities in their health system, and this has implications of funding for research and dissemination activities as well [39,40]. Many health systems (including health research) in sub-Saharan Africa are largely dependent on donor support. Whereas donor funding has reflected in beneficial outcomes of many interventions, such can also have a deleterious effect on the health system, as noted by stakeholders at the convening. Donors have been known to skew local research and policy agenda in favor of foreign interests [19,41]. Nigeria´s research agenda setting dates back to 1964 with the establishment of the National Council for Scientific and Industrial Research which was later restructured into four distinct research councils (the Agricultural Research Council of Nigeria; Medical Research Council of Nigeria; Natural Science Research Council of Nigeria; and Industrial Research Council of Nigeria) [42]. Many other policy-backed avenues toward increased government and industry investment for research have since followed but financial, governance and resource complexities have proven to be stumbling blocks. For example, a national health research agenda currently exists in Nigeria, but only 0.08% of the national health expenditure is allocated to research [19]. This is despite the country´s commitment to a 20% increase in budgetary support to health research institutions for research by 2022 [43]. The poor budgetary allocation and release of funds, despite this commitment, may be reflective of the need to for stronger involvement of HPSR in strategic and operational planning/ budgetary processes. This way, strategic plans may be more reflective of fiscal and political realities.

**Inequalities in research and policymaking:** inequalities remain a challenge in health systems across the world. Lots of the literature has emphasized inequalities in relation to user outcomes of the health system, for example health status, access and utilization of services [44-46]. It is, however, interesting that participants at the convening emphasized inequalities with respect to evidence generation and decision making, and how these affect health outcomes of the population. The two inequalities raised were gender and transgenerational inequalities. At the bottom line of these inequalities lie the much-discussed power asymmetries in the research and policymaking spaces [47-52]. Male dominance at the higher levels of decision making is not unique to Nigeria or sub-Saharan Africa. A recent study in Cambodia found that gender norms shape men´s and women´s career progression such that while favorably changing by allowing more women to enter medical education; there still exists significant barriers to female leadership in the sector. Females are rather dominant at the front lines [53]. Similarly, there have been discussions on youth participation in research and decision making. But these discussions have not significantly transitioned from mere rhetoric and high-level policies concerning the right of young people to participate in decision making, to its applied reality. Thus, there is often a discordance between the lived realities and priorities of young people, and the decisions made by policymakers who are mostly older people [54]. The enduring control of the participation agenda by adults is considered responsible for this discordance. This power asymmetry is particularly important in a country like Nigeria where the average age is 18 years (global average is 29 years) [55]. Charles and Haines have advocated participatory action research as a bridge to this trans generational gap in the translation of evidence to policy [54].

## Conclusion

Our study provides stakeholder perspectives on contextual and potentially effective KT strategies in Nigeria’s policy making space, which may apply to other similar jurisdictions in LMICs. Overall, better interaction among the various stakeholders will improve the evidence generation, translation and use in Nigeria. A road map for dissemination of findings from this unconference has been developed and is being implemented across the spectrum of the formal and informal strata of the health system.

### What is known about this topic

- The field of knowledge translation has sought answers to bridge the evidence-policy gaps that exist between researchers (knowledge producers) and policymakers (knowledge users) who (are expected to) use research-produced knowledge to make health policies;
- In low- and middle-income countries, support for evidence-informed healthcare has gained traction in recent years, but arguments have been put forward those approaches in evidence-based medicine would not work in public health policy making.

### What this study adds

- This study provides stakeholder perspectives on contextual and potentially effective KT strategies in Nigeria’s policy making space, which may apply to other similar jurisdictions in LMICs;
- Gender and generational inequalities can significantly impede evidence informed policymaking; government funding of research is a key determinant on the quality of evidence generated and used;
- Community participation in research and policymaking is essential; a framework for understanding gaps and potential bridging strategies was developed as an output of this study.

## References

[1] Eboreime EA (2019). Bridging the 'two communities': how an emerging primary healthcare global research consortium can help achieve universal health coverage in low and middle-income countries. BMJ Glob Health.

[2] Uneke CJ, Sombie I, Uro-Chukwu HC, Mohammed YG, Johnson E (2018). Promoting evidence informed policymaking for maternal and child health in Nigeria: lessons from a knowledge translation workshop. Health Promot Perspect.

[3] Gilson L, World Health Organization (2013). Health policy and system research: a methodology reader: the abridged version. World Health Organization.

[4] Lang ES, Wyer PC, Haynes RB (2007). Knowledge translation: closing the evidence-to-practice gap. Ann Emerg Med.

[5] Wellstead A, Cairney P, Oliver K (2018). Reducing ambiguity to close the science-policy gap. Policy Des Pract.

[6] Martin K, Mullan Z, Horton R (2019). Overcoming the research to policy gap. Lancet Glob Health.

[7] Price JA, Guinness L, Irava W, Khan I, Asante A, Wiseman V (2016). How to do (or not to do) translation of national health accounts data to evidence for policy making in a low resourced setting. Health Policy Plan.

[8] Cairney P, Oliver K (2017). Evidence-based policymaking is not like evidence-based medicine, so how far should you go to bridge the divide between evidence and policy?. Health Res Policy Syst.

[9] Head BW (2008). Three lenses of evidence-based policy. Australian Journal of Public Administration.

[10] Eboreime EA, Banke-Thomas A (2022). Beyond the Science: Advancing the “Art and Craft” of Implementation in the Training and Practice of Global Health. Int J Health Policy Manag.

[11] Eboreime EA, Iwu CJ, Banke-Thomas A (2020). Any and every cure for COVID-19: an imminent epidemic of alternative remedies amidst the pandemic?. Pan Afr Med J.

[12] Carley S, Horner D, Body R, Mackway-Jones K (2020). Evidence-based medicine and COVID-19: what to believe and when to change. Emerg Med J.

[13] Amaechi UA, Sodipo BO, Nnaji CA, Owoyemi A, Omitiran K, Okedo-Alex IN (2020). Social approaches to COVID-19 pandemic response: effectiveness and practicality in sub-Saharan Africa. PanAfrican Medical Journal.

[14] Altindis E (2022). Inequitable COVID-19 vaccine distribution and the intellectual property rights prolong the pandemic. Expert Rev Vaccines.

[15] Ashraf MA, Muhammad A, Shafiq Y (2021). The Politics of COVID-19 Vaccine Distribution and Recognition. Public Health Rev.

[16] Lanziotti VS, Bulut Y, Buonsenso D, Gonzalez-Dambrauskas S (2022). Vaccine apartheid: This is not the way to end the pandemic. J Paediatr Child Health.

[17] Federal Government of Nigeria (2018). Second National Strategic Health Development Plan (2018 - 2022).

[18] Defor S, Kwamie A, Agyepong IA (2017). Understanding the state of health policy and systems research in West Africa and capacity strengthening needs: scoping of peer-reviewed publications trends and patterns 1990-2015. Health Res Policy Syst.

[19] Okedo-Alex IN, Akamike IC, Olisaekee GO, Okeke CC, Uneke CJ (2021). Identifying advocacy strategies, challenges and opportunities for increasing domestic health policy and health systems research funding in Nigeria: Perspectives of researchers and policymakers. Health Res Policy Syst.

[20] Uneke CJ, Sombie I, Johnson E, Uneke BI (2020). Lessons Learned from Strategies for Promotion of Evidence-to-Policy Process in Health Interventions in the ECOWAS Region: A Rapid Review. Niger Med J.

[21] Berman J, Mitambo C, Matanje-Mwagomba B, Khan S, Kachimanga C, Wroe E (2015). Building a knowledge translation platform in Malawi to support evidence-informed health policy. Health Res Policy Syst.

[22] Jessani NS, Hendricks L, Nicol L, Young T (2019). University Curricula in Evidence-Informed Decision Making and Knowledge Translation: Integrating Best Practice, Innovation, and Experience for Effective Teaching and Learning. Front Public Health.

[23] Turner MW, Bogdewic S, Agha E, Blanchard C, Sturke R, Pettifor AS (2021). Learning needs assessment for multi-stakeholder implementation science training in LMIC settings: findings and recommendations. Implement Sci Commun.

[24] Systems Development Initiative (2020). HSGAbuja 2020 Convening: Strengthening Capacity for Health Policy Systems Research in Nigeria.

[25] Braun V, Clarke V (2006). Using thematic analysis in psychology. Qualitative Research in Psychology.

[26] Uneke CJ, Ezeoha AE, Uro-Chukwu HC (2018). Promoting evidence-informed policymaking through capacity enhancement in implementation research for health researchers and policymakers in Nigeri: A cross-sectional study. J Educ Health Promot.

[27] Uneke CJ, Ezeoha AE, Uro-Chukwu HC, Ezeonu CT, Igboji J (2018). Promoting Researchers and Policy-Makers Collaboration in Evidence-Informed Policy-Making in Nigeria: Outcome of a Two-Way Secondment Model between University and Health Ministry. Int J Health Policy Manag.

[28] Uneke CJ, Sombie I, Johnson E, Uneke BI, Okolo S (2020). Promoting the use of evidence in health policymaking in the ECOWAS region: the development and contextualization of an evidence-based policymaking guidance. Global Health.

[29] Hawkes S, Aulakh KB, Jadeja N, Jimenez M, Buse K, Anwar I (2016). Strengthening capacity to apply health research evidence in policy making: experience from four countries. Health Policy Plan.

[30] Eboreime EA, Olawepo JO, Banke-Thomas A, Abejirinde IO, Abimbola S (2021). Appraising and addressing design and implementation failure in global health: A pragmatic framework. Glob Public Health.

[31] Drieghe L, Orbie J, Potjomkina D, Shahin J (2022). Participation of civil society in EU trade policy making: how inclusive is inclusion. New Political Econ.

[32] Palinkas LA, Saldana L, Chou CP, Charnberlain P (2017). Use of Research Evidence and Implementation of Evidence-Based Practices in Youth-Serving Systems. Child Youth Serv Rev.

[33] Nyirenda D, Sariola S, Gooding K, Phiri M, Sambakunsi R, Moyo E (2018). We are the eyes and ears of researchers and community: Understanding the role of community advisory groups in representing researchers and communities in Malawi. Dev World Bioeth.

[34] Eboreime EA, Idika O, Omitiran K, Eboreime O, Ibisomi L (2019). Primary healthcare planning, bottleneck analysis and performance improvement: An evaluation of processes and outcomes in a Nigerian context. Eval Program Plann.

[35] Eboreime EA, Nxumalo N, Ramaswamy R, Ibisomi L, Ihebuzor N, Eyles J (2019). Effectiveness of the Diagnose-Intervene-Verify-Adjust (DIVA) model for integrated primary healthcare planning and performance improvement: an embedded mixed methods evaluation in Kaduna state, Nigeria. BMJ Open.

[36] Eboreime EA, Olawepo JO, Banke-Thomas A, Ramaswamy R (2021). Evaluating the design and implementation fidelity of an adapted Plan-Do-Study-Act approach to improve health system performance in a Nigerian state. Eval Program Plann.

[37] Banke-Thomas A, Wong KL, Collins L, Olaniran A, Balogun M, Wright O (2021). An assessment of geographical access and factors influencing travel time to emergency obstetric care in the urban state of Lagos, Nigeria. Health Policy Plan.

[38] Jones T, Ho L, Kun KK, Milsom P, Shakpeh J, Ratnayake R (2018). Rebuilding people-centred maternal health services in post-Ebola Liberia through participatory action research. Glob Public Health.

[39] Shroff ZC, Javadi D, Gilson L, Kang R, Ghaffar A (2017). Institutional capacity to generate and use evidence in LMICs: current state and opportunities for HPSR. Health Res Policy Syst.

[40] Grépin KA, Pinkstaff CB, Shroff ZC, Ghaffar A (2017). Donor funding health policy and systems research in low-and middle-income countries: how much, from where and to whom. Health Res Policy Syst.

[41] Eboreime EA, Abimbola S, Obi FA, Ebirim O, Olubajo O, Eyles J (2017). Evaluating the sub-national fidelity of national Initiatives in decentralized health systems: Integrated Primary Health Care Governance in Nigeria. BMC Health Serv Res.

[42] Kuhlmann S, Ordóñez-Matamoros G (2017). Research handbook on innovation governance for emerging economies: towards better models. Edward Elgar Publishing.

[43] Federal Ministry of Health Nigeria (2018). Second National strategic health development plan (NSHDP II).

[44] Eboreime E, Abimbola S, Bozzani F (2015). Access to Routine Immunization: A Comparative Analysis of Supply-Side Disparities between Northern and Southern Nigeria. Glob Health Action.

[45] Arcaya MC, Arcaya AL, Subramanian SV (2015). Inequalities in health: definitions, concepts, and theories. Glob Health Action.

[46] McCartney G, Popham F, McMaster R, Cumbers A (2019). Defining health and health inequalities. Public Health.

[47] Gore R, Parker R (2019). Analysing power and politics in health policies and systems. Glob Public Health.

[48] Clavero S, Galligan Y (2021). Delivering gender justice in academia through gender equality plans. Normative and practical challenges. Gender Work Organ.

[49] Engstrand AK (2019). From quotas of men to gender mainstreaming: gender equality policies in academia from the 1960s to the 2000s. InNordic Gender Equality Policy in a Europeanisation Perspective.

[50] Keisu BI, Abrahamsson L, Ronnblom M (2015). Entrepreneurship and gender equality in academia: a complex combination in practice. Nord J Working Life.

[51] Winchester HPM, Browning L (2015). Gender equality in academia: a critical reflection. J High Educ Policy M.

[52] Hawkins R, Manzi M, Ojeda D (2014). Lives in the making: power, academia and the everyday. ACME: An Int J Geogr.

[53] Vong S, Ros B, Morgan R, Theobald S (2019). Why are fewer women rising to the top? A life history gender analysis of Cambodia's health workforce. BMC Health Serv Res.

[54] Charles A, Haines K (2014). Measuring young people´s participation in decision making. The Int J Child Rights.

[55] Reed HE, Mberu BU (2014). Capitalizing on Nigeria's demographic dividend: reaping the benefits and diminishing the burdens. Etude Popul Afr.

